# Effect of Ultrasound-Treated Arabinoxylans on the Oxidative Stability of Soybean Oil

**DOI:** 10.3390/antiox9020147

**Published:** 2020-02-10

**Authors:** Mayra A. Mendez-Encinas, Elizabeth Carvajal-Millan, Jesús Ortega-García, Lubitza B. Santiago-Gómez, Yubia De Anda-Flores, Karla G. Martínez-Robinson, Dora E. Valencia-Rivera

**Affiliations:** 1 Biopolymers, Research Center for Food and Development (CIAD), Hermosillo, Sonora 83304, Mexico; ecarvajal@ciad.mx (E.C.-M.); yubia.deanda@estudiantes.ciad.mx (Y.D.A.-F.); karlagm@ciad.mx (K.G.M.-R.); 2 Department of Chemical Biological and Agropecuary Sciences, University of Sonora, Caborca, Sonora 83621, Mexico; jesus.ortega@unison.mx (J.O.-G.); lubitza.santiago@gmail.com (L.B.S.-G.)

**Keywords:** arabinoxylan, ultrasonication, antioxidant capacity, rancimat method, oxidative stability

## Abstract

Arabinoxylans (AX) are polysaccharides with antioxidant activity and emulsifying properties, which make them an attractive alternative for its potential application as a natural antioxidant in oils. Therefore, this work aimed to investigate the effect of ultrasonic treatment of AX on their antioxidant capacity and its ability to improve the oxidative stability of soybean oil. For this purpose, AX were exposed to ultrasonic treatment at 25% (100 W, AX-1) and 50% (200 W, AX-2) power and an operating frequency of 20 KHz during 15 min, and their macromolecular properties (weight average molecular weight (Mw), polydispersity index and intrinsic viscosity) were evaluated. The antioxidant capacity of AX was determined by the DPPH assay and Rancimat test. Results showed that ultrasonic treatment did not affect the molecular identity of the polysaccharide but modified its Mw distribution. AX-1 showed the highest antioxidant activity (75% inhibition) at 533 µg/mL by the DPPH method compared to AX and AX-2. AX at 0.25% (*w*/*v*) and AX-1 at 0.01% (*w*/*v*) exerted the highest protective effects on oxidative stability of soybean oil with induction periods of 7.69 and 5.54 h, respectively. The results indicate that AX could be a good alternative for the potential application as a natural antioxidant in oils.

## 1. Introduction

Lipid oxidation is one of the most common drawbacks for the oil industry as it affects the shelf life and quality of oils and fats [1]. Oxidative lipid degradation affects negatively the organoleptic attributes of oils by causing unfavorable changes in odor, taste, and appearance, and also decreasing its nutritional value [2,3]. For several years, synthetic antioxidants have been extensively used by food industries in order to retard lipid oxidation and preserve the quality standards of oils. Among synthetic antioxidants, butylated hydroxytoluene (BHT), butylated hydroxyanisole (BHA), and tert-butylhydroquinone (TBHQ) are routinely used in food industry as food protectants due to their good oxidative stability and low cost [1]. However, several recent studies have reported their potential negative effects on health due to their toxicity and carcinogenicity [4,5]. In fact, the use of some antioxidants such as TBHQ is not allowed for food applications in Canada, Japan, and Europe [5,6]. In this regard, efforts to minimize or replace the use of synthetic antioxidants in foods have increased considerably in the last years. There is a growing demand for discovering antioxidants of natural origin as a new alternative to protect foods from lipid deterioration. Particularly, plant-based antioxidants have been of great interest due to their high content of antioxidant compounds such as phenolic acids, tannins, flavonoids, and more [7].

Agro-industrial by-products represent an attractive alternative for the production of compounds with potential antioxidant capacity. Distiller’s dried grains with solubles (DDGS), which is the main co-product from dry-grind corn ethanol production represent a good source of arabinoxylans (AX). AX are non-starch polysaccharides with antioxidant capacity [8] and a structure consisting of a linear β-(1–4)-xylose backbone with some arabinose substitutions at O-3 and/or O-2 positions. Additionally, ferulic acid (FA) residues can be ester-linked to some arabinose units at O-5 position [9]. Other components such as sugars (galactose, glucose, mannose, glucuronic acid), hydroxycinnamic acids (coumaric and sinapic acid) and protein may be part of its structure [10,11]. AX exert antioxidant ability which has been widely related to its phenolic content, mainly FA as it is the most abundant into its structure [8,12,13]. Moreover, health-promoting properties of AX such as prebiotic, anti-obesogenic, immunomodulating and antiproliferative properties have been previously documented [14,15,16,17]. AX have also the capacity to stabilize oil-in-water dispersions due to the protein moiety associated with its structure [18]. Perhaps, its emulsifying properties can be compared with Arabic gum, a well-known commercial stabilizer [19]. Then, AX appear to be an attractive alternative for its potential application as a natural antioxidant in oils.

It is known that the structural features of AX influence on their functional and biological properties. Among the molecular characteristics of AX, its molecular weight (Mw) appears to be an important factor affecting its antioxidant capacity as has been reported by several studies [20,21,22]. Malunga and Beta [23] found that a decrease in the Mw of AX improved the antioxidant activity of the molecule. Conversely, a positive correlation between higher Mw and a higher degree of polymerization with antioxidant capacity has been documented [21,22]. Previously, Li et al. [20] reported that the use of ultrasonic treatment in AX reduced its Mw and increased its antioxidant capacity due to an increase in the concentration of phenolic compounds. Although, the potential application of plant extracts as natural antioxidants in oils has been widely investigated, there are no reports about the effect of AX on the oxidative stability of oils. Therefore, the aim of this work was to evaluate the effect of ultrasonic treatment of AX on their macromolecular and antioxidant properties and its effectiveness promoting the oxidative stability of soybean oil.

## 2. Materials and Methods

### 2.1. Materials

AX were previously extracted from DDGS and characterized as described elsewhere [10]. AX presented 70% dry basis (d.b) of pure AX (sum arabinose + xylose), 3.36% glucose, 4.20% galactose, a protein content of 7.20%, and an arabinose to xylose ratio (A/X ratio) of 0.7. Antioxidant-free soybean oil was obtained from the Mexican oil industry “Derivados de Oleoginosas del Valle” located in Obregon, Mexico. All chemical products were purchased from Sigma Chemical Co. (St. Louis, MO, USA).

### 2.2. Methods

#### 2.2.1. Ultrasonic Treatment of AX

AX dispersions (5 mg/mL) in water were prepared and exposed to different ultrasonic treatments. An Ultrasonic Homogenizer Omni Sonic Ruptor 400 (Omni International, Inc., Kennesaw, GE, USA) with a 5/32″ probe was used. AX were exposed to two conditions: AX-1, 25% power (100 W) and 15 min homogenization time (5 min cycles), and AX-2, 50% power (200 W) and 15 min homogenization time (5 min cycles) at an operating frequency of 20 KHz. Samples were frozen and lyophilized at −46 °C/0.18 mbar during 24 h (Labconco, Kansas, MO, USA).

#### 2.2.2. Physicochemical Properties

Weight average molecular weight (*Mw*), intrinsic viscosity ([*η*]) and polydispersity index (*PI* = *Mw*/*Mn*) of samples were determined using a size-exclusion high-performance liquid chromatography (SE-HPLC, Wyatt Technology Corp., Santa Barbara, CA, USA) system with a Dawn HELEOS-II 8 multi-angle laser light scattering (MALS) detector coupled with a ViscoStar-II viscosimeter and an Optilab T-rex refractive index detector according to Dervilly-Pinel et al. [24]. In brief, samples were dissolved in 50 mM NaNO_3_/0.02% NaN_3_ buffer (5 mg/mL) at 80 °C for 1 h and centrifuged at 15,000× *g* and 20 °C for 10 min. Then, samples were filtered with a 0.45 µm pore filter (Millipore) and injected. The flow rate was 0.7 mL/min, and a 50 mM NaNO_3_/0.02% NaN_3_ buffer was used as eluent. The SE-HPLC system consisted of two columns, Shodex OH-pack SB HQ 804 and 805 (Shodex Showa Denco K.K., Tokyo, Japan) and a chromatographic system (Agilent Technologies, Inc., Santa Clara, CA, USA). The *Mw*, [*η*] and *PI* values were calculated using the software ASTRA 6.1. (Wyatt Technology Corp., Santa Barbara, CA, USA). A specific refractive index increment (dn/dc) value of 0.146 mL/g was used.

#### 2.2.3. Fourier Transform Infrared Spectroscopy Analysis

AX samples were recorded on a Nicolet iS50 FT-IR spectrometer (Thermo Fisher Scientific Inc., Waltham, MA, USA) in absorbance mode from 400 to 4000 cm^−1^ range at 20 °C. Thirty-two scans at a resolution of 4 cm^−1^ were averaged and referenced against air [8].

#### 2.2.4. Ferulic Acid Content

FA was quantified by RP-HPLC as previously reported [25,26] after a de-esterification step. The de-esterification step was omitted for the free FA content determination. An Alltima C18 column (250 mm 4.6 mm; Alltech Associates, Inc., Deerfield, IL, USA) and a photodiode array detector (Waters 996; Waters Corporation, Milford, MA, USA) were used. Detection was followed by UV absorbance at 320 nm.

#### 2.2.5. Total Phenolic Content

The total phenolic content was carried out using the Folin-Ciocalteau colorimetric assay according to Popova et al. 2004 [27] with slight modifications [28,29]. In brief, a volume of 10 µL sample (10 mg/mL) was placed into a 96-well microplate followed by 60 µL of sodium carbonate (7% *w*/*v*), 40 µL of Folin–Ciocaltau reagent (0.2 N) and 90 µL of distilled water. The mixture reaction was kept in the dark for 1 h at 25 °C, and absorbance was measured at 750 nm using a microplate spectrophotometer (Thermo Scientific MultiSkan Go, Madrid, Spain). Results were expressed as mg gallic acid/g dried sample (mg EGA/g) by a dose-response calibration curve of gallic acid. Each assay was carried out by triplicate.

#### 2.2.6. Radical Scavenging Capacity

The radical scavenging activity was determined using the DPPH method according to Malunga and Beta [30] with slight modifications. Different concentrations of AX in ultrapure water (0–2000 µg/mL) were prepared. Aliquots of 400 µL of each AX solution were mixed with 350 µL of absolute methanol. Then, 100 µL of each solution was placed into a 96-well microplate and 100 µL of DPPH solution (45 mM, methanol/water) was added and mixed. The mixture was left in the dark for 30 min and absorbance was measured at 517 nm using a microplate spectrophotometer (Thermo Scientific MultiSkan Go, Madrid, Spain). Absolute methanol was used as blank. The antioxidant capacity was reported as percentage inhibition of the DPPH radical and was calculated using the following formula:

(1) $$I=\frac{A_{0}-A}{A_{0}}\;\times \;100$$

where *I* = % DPPH inhibition, *A*_0_ = absorbance of blank (*t* = 0 h) and *A* = absorbance of sample (*t* = 30 min).

The half minimal effective concentration (EC_50_), which corresponds to the amount of sample required to neutralize 50% of DPPH radical was calculated from the % inhibition curve. In addition, EC_50_ values were expressed as µmol of Trolox (6-hydroxy-2,5,7,8-tetramethylchoman-2-carboxylic acid) equivalent capacity per gram of sample (µmol TEAC/g) using a dose-response curve of Trolox.

#### 2.2.7. Oxidative Stability of Oils by Rancimat Method

Samples of commercial soy oil (3.5 g) containing different concentrations of AX (0.25%, 0.1% and 0.01%) were placed into the reaction cells and exposed to oxidation at 110 °C and air-flow 20 L/h. The induction period (IP, h) of samples was automatically measured by the equipment and corresponded to the time when the sample begins its auto-oxidation and generates polar compounds that can produce changes in conductivity. An increase in the IP indicated the antioxidant ability of sample, while a decrease in the IP indicated oxidative ability. TBHQ and tocopherol (TP) were used as a reference for synthetic and natural antioxidants, respectively. TBHQ and TP were used at a concentration of 0.02% (concentration used in the food industry). Soybean oil without any additive (antioxidant-free) was used as control.

#### 2.2.8. Statistical Analysis

Results are expressed as mean ± SD values. Data were analyzed using one-way analysis of variance (ANOVA) with Tukey-Kramer test (NCSS, 2007, NCSS, LLC, Kaysville, TN, USA).

## 3. Results and Discussion

### 3.1. Effect of Ultrasonic Treatment on the Macromolecular Features of AX

**Figure 1:**
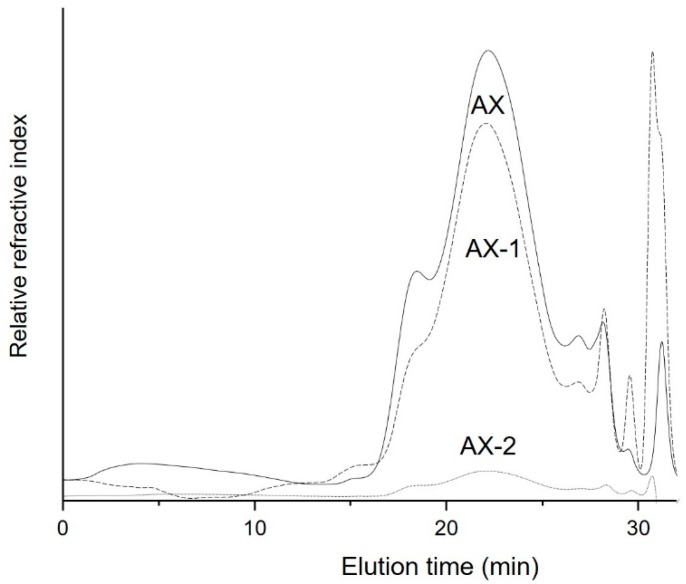
SE-HPLC (size-exclusion high-performance liquid chromatography) chromatograms of AX treated with different ultrasonic conditions. AX = untreated AX, AX-1 = AX treated with 25% power for 15 min, AX-2 = AX treated with 50% power for 15 min.

Several studies have documented that low-Mw polysaccharides exert better biological properties than un-degraded or high-Mw polysaccharides [23,31,32]. Molecules with low Mw have better water-solubility, lower viscosity and simple structure [33]. The ultrasonic treatment has been widely used for the degradation of polysaccharides in order to reduce their Mw and improve their biological activities [20,32,34]. Therefore, in this study, AX were exposed to ultrasonic treatment at 25% power (AX-1) and 50% power (AX-2) for 15 min and the effect of such treatments on their macromolecular features are presented in Table 1. The ultrasonic treatment reduced the Mw from 598 to 476 kDa in AX-1. A previous study observed a similar behavior where Mw of AX decreased from 820 to 581 kDa as ultrasonic treatment time increased from 0 to 45 min and the power increased from 0 to 480 W [20]. Interestingly, AX-2, which were exposed to a higher ultrasonic power showed a higher Mw (744 kDa) than that obtained for AX-1. However, it should be noticed that the Mw corresponds to specific fractions of the polysaccharide population. The chromatograms show that the high Mw fractions were almost completely degraded in AX-2 and only one small fraction of high Mw (744 kDa) remained un-degraded after the ultrasonic treatment (Figure 1). Thus, the chromatographic results suggest that the high Mw fractions of AX were degraded to lower Mw molecules (possibly oligosaccharides) which were not detected during the analysis. Conversely, the elution profile patterns of AX-1 and AX are close similar; only a slight reduction in a high Mw fraction (17 min) was observed which possibly was degraded to produce low Mw chains as evidenced by the increase in the peak corresponding to a lower Mw fraction (32 min).

PI values for AX samples ranged from 1.82 to 1.75, which are similar to those reported for other AX isolated from DDGS (1–1.8) [12]. The PI value of AX-1 (1.82) was not dramatically affected by the ultrasonic treatment as it was similar to that obtained for untreated AX (1.85). This indicates that the low ultrasonic power (25%) used in AX-1 did not depolymerize the AX backbone to a high level as evidenced by the elution profile pattern, which was closely similar to that of AX (Figure 1). On the contrary, the PI value for AX-2 was 1.75 which agrees with the decrease of high Mw polysaccharide fractions and the possible formation of lower Mw fractions, indicating an evident depolymerization of the polysaccharide. Viscosity is strongly associated with the polymer chain length and conformation of the polymer [33]. According to the results, ultrasonic treatment was able to decrease the [η] of AX from 13 to 11 dL/g (Table 1). This slight decrease could be mainly attributed to the ultrasonic conditions used in the experiment. Wang et al. [33] indicated that higher ultrasonic power and longer times are required to break-up the polysaccharide chains or glycosidic bonds than to break the polymer aggregates. These authors were able to reduce the [η] from 15 to 0.5 dL/g by increasing the ultrasonic power from 0% to 70%. Therefore, the ultrasonic treatment used in AX-1 (25% power) could be able to break-up the polymer aggregates and only small fractions of high Mw polysaccharide chains as evidenced by a slight decrease in the [η]. Although the higher ultrasonic power used in AX-2 was able to degrade polysaccharide chains, the small fraction of high Mw, which remained after the treatment, could be contributing to its viscosity.

The effect of ultrasonic treatment on the FA content of AX was also evaluated. AX presented a FA content of 6.72 µg/mg AX, indicating a highly ferulated structure. Similar FA contents ranging from 5.45 to 7.53 µg/mg AX have been previously reported for other AX extracted from DDGS [8,10]. According to the results, the FA content in AX-1 and AX-2 was not affected by the ultrasonic treatment, indicating that the treatment was not able to release the FA residues from the polysaccharide chains. This result agrees with the free-FA determination as no free-FA was identified in AX-1 nor AX-2 during the analysis (data are not shown).

### 3.2. FT-IR Analysis

**Figure 2:**
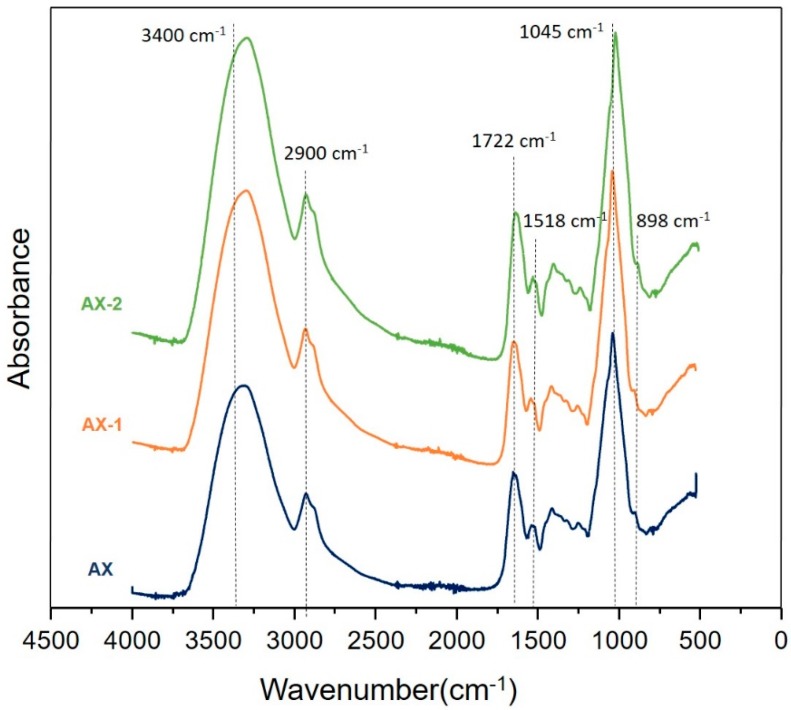
FT-IR spectra of AX treated with ultrasonication. Untreated AX (AX), AX treated at 25% power for 15 min (AX-1), and AX treated at 50% power for 15 min (AX-2).

FT-IR analysis of AX samples was performed in order to identify the molecular identity of AX after the ultrasonic treatment based on typical spectral patterns and bands reported for this polysaccharide [12,35]. The untreated and treated AX spectra are shown in Figure 2. All three samples showed a similar pattern which indicates that the ultrasonic treatment did not have a significant effect on the chemical structure of the polysaccharide. The spectra showed a broad band between 1200–1800 cm^−1^, which is typical for polysaccharides such as AX [35]. Particularly, the bands detected at 1045 and 898 cm^−1^ are related to the antisymmetric C–O–C stretching mode of the β-(1–4)-glycosidic linkages of the AX backbone [35]. A loss of peak multiplicity in the region between 1120–1200 cm^−1^ has been attributed to a highly substituted AX which agrees with the high A/X ratio (0.7) of AX used in this study [8,35]. Moreover, the ultrasonic treatment did not modify the A/X ratio of AX as evidenced by the loss of peak multiplicity observed in the AX-1 and AX-2 spectral patterns. The presence of the bands at 3400 cm^−1^ and 2900 cm^−1^ which are associated with the OH stretching and CH_2_ groups, respectively corresponds to the fingerprint region of polysaccharides related to AX [36,37]. The absorbance bands identified at 1722 cm^−1^ (ester bond, C=O) and 1518 cm^−1^ (aromatic vibration ring) are related to the presence of phenolic acids such as FA [38]. These findings suggest that the ultrasonic treatment applied to AX did not modify the molecular identity of the polysaccharide, but it could affect its physicochemical and functional properties. Similarly, Li et al. [20] found that ultrasonic treatment (120–480 W, 15–45 min) on AX did not have a significant effect on the main absorbance bands.

### 3.3. Total Phenolic Content and Antioxidant Capacity

**Figure 3:**
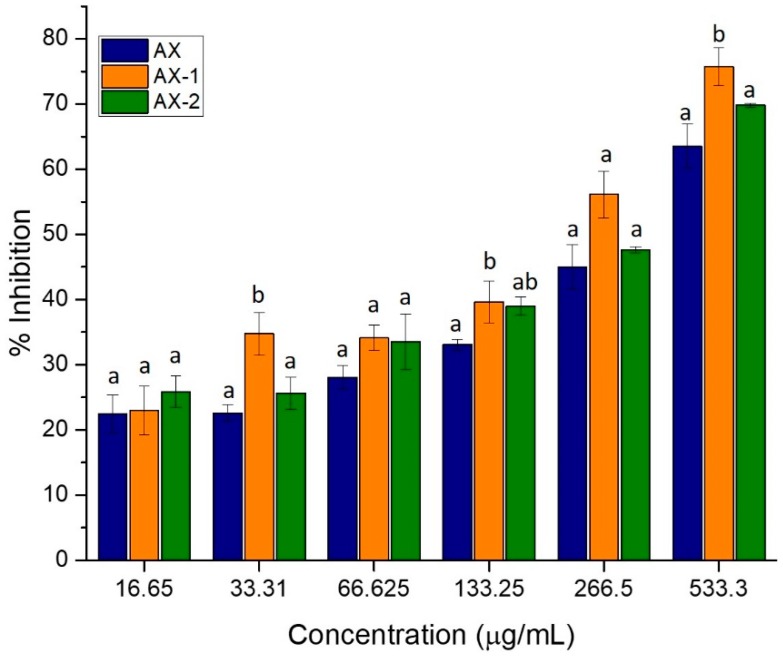
Effect of different conditions of ultrasonic treatment on the antioxidant activity of AX. Untreated AX (AX), AX treated at 25% power for 15 min (AX-1), and AX treated at 50% power for 15 min (AX-2). Values are presented as means ± SD of three replicates. Different letter (a,b) within the same group of concentration indicates a significant difference (*p* < 0.05).

The ability of AX to scavenge free radicals was evaluated by the DPPH method. Figure 3 shows the antioxidant capacity of AX exposed to different ultrasonic conditions. The antioxidant capacity of AX samples increased as the concentration increased following a dose-dependent behavior. The higher antioxidant capacity was observed at a concentration of 533.3 µg/mL with 75%, 69%, and 63% inhibition for AX-1, AX-2, and AX, respectively. EC_50_ values, which correspond to the concentration of AX required to scavenge 50% of the DPPH radical were also determined and are shown in Table 2. According to the results, the ultrasonic treatment of AX favored the antioxidant activity of the polysaccharide by reducing their EC_50_ values. AX-1 presented the lower EC_50_ value (225.17 µg/mL) which agrees with the highest antioxidant capacity detected for this sample (75%). In a previous study, Li et al. [20] observed that the antioxidant capacity of AX increased as the ultrasonic power and the time increased and the Mw decreased. However, in this study, AX treated with low ultrasonic power (AX-1, 25%) showed a higher antioxidant capacity than AX treated with higher ultrasonic power (AX-2, 50%). It has been documented that at low ultrasonic power (20% amplitude), the shear force of cavitation may be only sufficient to break-up the polysaccharide aggregates, but not to degrade the polysaccharide chains [33]. Therefore, it could be possible that the low ultrasonic power used in AX-1 (25%) allowed the breaking of aggregates and helped to extend the polysaccharide chains. Thus, the higher antioxidant capacity observed in AX-1 could be related to the higher amount of FA residues exposed after the ultrasonic treatment which were available to exert the scavenging activity. Antioxidant capacity of AX has been mainly attributed to the presence of FA in its structure as reported elsewhere [12,13]. On the contrary, although a considerable decrease in the high Mw fractions of AX-2 was observed, the remaining high Mw fraction could be contributing to a decrease in the antioxidant capacity of the polysaccharide. The possible formation of aggregates between the high Mw polymer chains may make the phenolic acid residues less accessible and affect its antioxidant capacity. AX chains can be cross-linked through the coupling of FA residues to form dimers of FA (di-FA) which act as covalent bridges between the polysaccharide chains [39]. The ultrasonic treatment was not able to cleave the FA residues from AX chains as the amount of FA was preserved after the treatment (Table 1). Thus, the high Mw fractions observed in the SE-HPLC chromatogram of AX-2 (Figure 1) could be related to the covalent cross-linked AX chains which were not degraded by the ultrasonic treatment.

The antioxidant capacity of AX was also expressed as Trolox equivalent (TEAC) for comparison purposes (Table 2). Previous studies have obtained a similar antioxidant capacity (32 µmol TEAC/g AX) for AX from maize using the DPPH method [8,13]. These authors have attributed the antioxidant capacity of the polysaccharide to its FA content. The presence of electron-donating groups on the benzene ring of FA confers it the ability of terminating free radical reactions. FA donates a hydrogen atom to form a highly resonance stabilized phenoxy radical which exerts the antioxidant effect [40]. The ultrasonic treatments increased the antioxidant capacity of AX by 7–12%, being AX-1 the treatment with the highest antioxidant activity (44 µmol TEAC/g AX). A positive correlation between total phenolic content and antioxidant capacity of AX was observed by Herrera-Balandrano et al. [13]. However, the ultrasonic treatment did not show a significative effect on the amount of total phenolic compounds of AX (Table 2), suggesting that the amount of total phenolic compounds did not influence the antioxidant activity of the polysaccharide. Several studies have reported that a decrease in the Mw could improve the antioxidant activity of polysaccharides [20,32,34]. However, based on these findings, the Mw appears not to be the main factor influencing the antioxidant activity of AX under the conditions tested. Then, the conformational structure of the polysaccharide could also be affecting such biological property.

### 3.4. Oxidative Stability of Soybean Oil

**Figure 4:**
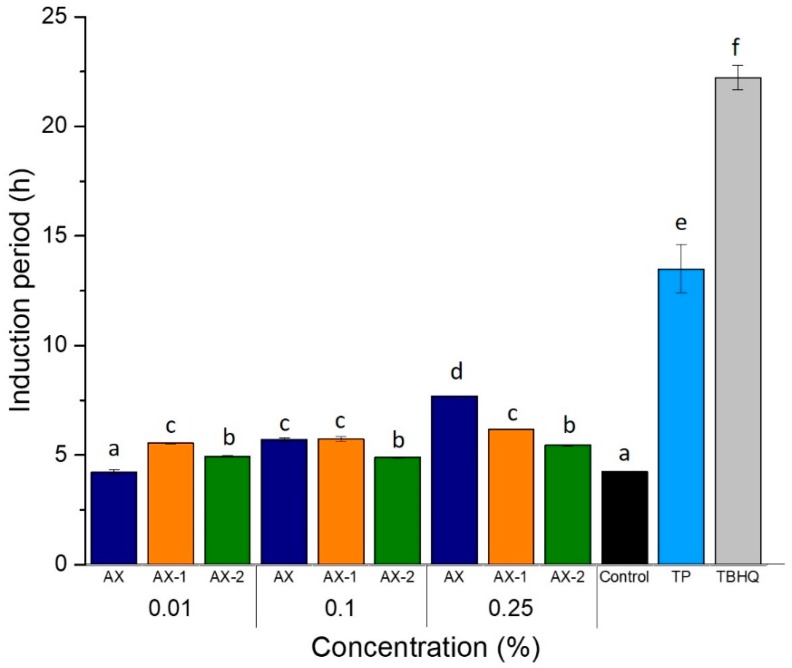
Oxidative stability of soybean oil treated with different concentrations of AX, AX-1, AX-2 (% *w*/*v*), tocopherol (TP, 0.02% *w*/*v*) and tertbutylhydroquinone (TBHQ, 0.02% *w*/*v*) at 110 °C and air-flow rate of 20 L/h. Antioxidant-free soybean oil was used as Control. Results are expressed as means ± SD of three replicates. Different letters within the same group of concentration indicate statistical difference (*p* < 0.05) when compared with Control.

The Rancimat test is a helpful technique used to determine the oxidative stability of fats and oils by measuring the changes in conductivity caused by volatile acids produced during accelerated oxidation of oils [41]. The time that elapses until the secondary products are detected is called induction period (IP) and indicates the resistance of oil to oxidation [1]. AX were evaluated for their potential to inhibit the accelerated autooxidation of soybean oil, and results are shown in Figure 4. The effect of synthetic (TBHQ) and natural (TP) antioxidants were also tested for comparison purposes. In general, all concentrations of either untreated or ultrasound-treated AX showed a protective effect on the oxidative stability of soybean oil, except for AX at 0.01% which exhibited a similar IP (4.23 h) than Control (antioxidant-free soybean oil). According to the results, the ultrasonic treatment favored the oxidative stability of oil when added at the lowest concentration tested (0.01%) as AX-1 showed the highest stabilizing effect with an IP of 5.54 h. On the contrary, AX exhibited the better antioxidant activity at the highest concentration evaluated (0.25%), showing an IP of 7.69 h, while AX-2 showed the lowest IP (5.45 h) at the same concentration. This behavior could be related to the effect of ultrasonic treatment on the conformational structure (secondary and tertiary structures) of the polysaccharide. It is well known that AX have a protein moiety attached to its structure which plays a very important role in the emulsifying properties of the polysaccharide [18]. The amphipathic nature of AX promotes the stabilization of oil-in-water emulsions [18]. Thus, the stabilization of polysaccharide chains could promote the interaction of oil with the phenolic compounds of AX and exert its antioxidant capacity. On the other hand, AX aggregates may be held by inter- and intra-molecular forces (hydrogen bonds, hydrophobic, van der Waals) limiting the accessibility to FA residues. Thus, when AX are exposed to ultrasonic treatment, the cavitation forces weakens the molecular forces between the polysaccharide chains causing the disruption of aggregates and leading to full exposure of phenolic acids. In addition, the application of higher ultrasound power to AX is able to break-up the polymer chains through the degradation of glycosidic linkages, which contributes to improve its solubility in water [33]. The ultrasonic treatment in AX-1 allowed to break-up the polymer aggregates and to expose the phenolic residues (FA) of the polysaccharide by promoting its antioxidant capacity. However, the treatment applied to AX-1 was also able to degrade some polysaccharide chains resulting in the production of a low Mw fraction (Figure 1). This fraction could contribute to reduce the stability of AX-1 in the lipidic system due to an increase in the amount of reducing sugars (OH) in the sample which finally affected its antioxidant ability. Therefore, a lower IP (6.17 h) was observed for AX-1 in comparison to AX (7.69 h). Interestingly, the ultrasonic treatment in AX-2 increased its water-solubility but decreased its solubility in oil. The ultrasonic treatment in AX-2 produced polysaccharide chains of lower Mw which increased the number of reducing sugars (hydroxyl groups). Thus, the highly hydrophilic character of the molecule affected its emulsifying capacity and also its antioxidant capacity. The increasing solubility of AX-2 was also evidenced visually when the sample was completely dissolved in water to produce a clear solution, but when added to soybean oil, a cloudy appearance was observed, indicating its low solubility in oils. Thus, the low solubility of AX-2 in oils may be affecting its antioxidant ability by decreasing the interaction between polysaccharide chains and oil. Moreover, it could be possible that the ultrasonic treatment applied to AX could affect its emulsifying properties by removing the protein moiety from the polysaccharide.

Table 3 shows the protection factors which are defined as the ratio of IP of the oil containing the test material (antioxidant) and the IP of pure oil without any additive [42]. The TBHQ and TP showed the most efficient antioxidant activity as they had higher protective factors, 5.24 and 3.18, respectively. The protection factor (PF) for AX samples ranged between 1.01 and 1.83. A higher PF value indicates a better protective effect of the sample tested on the oxidative stability of the oil. According to the results, all the samples had a protective effect against the oil autooxidation. The treatment of soybean oil with 0.25% AX exerted a better protective effect in soybean oil and improved its oxidative stability as evidenced by the highest IP (1.83). Lower PF values for whole barley extracts (1.31 to 1.59) and free FA (0.97) at higher concentrations (2% *w*/*w*) on corn oils have been previously reported [42]. These findings indicate the potential application of AX as a natural antioxidant in soybean oil.

## 4. Conclusions

The effect of ultrasonic treatment on the antioxidant properties of AX and its effectiveness in improving the oxidative stability of soybean oil was evaluated. Results showed that low-power ultrasonic treatment improved the antioxidant capacity of AX by the DPPH method when compared to high-power ultrasonic treatment. Moreover, the Rancimat test demonstrated that the addition of 0.25% (*w*/*v*) AX to soybean oil was the most effective treatment to retard oil rancidity. The findings also suggest that the conformational structure (degradation of polymer aggregates) of ultrasound-treated AX contributed to the antioxidant capacity of AX. Finally, the use of AX could be a good alternative as a natural antioxidant with health-promoting properties.

## Figures and Tables

**Table 1:** Macromolecular features of arabinoxylans (AX).

Sample	Mw	PI	[η]	FA ^1^
	kDa		dL/g	µg/mg AX
AX	598	1.82	13	6.72 ± 0.07
AX-1	476	1.85	11	6.49 ± 0.01
AX-2	744	1.75	12	6.24 ± 0.28

^1^ Values are presented as means ± SD of duplicates. Mw: molecular weight; PI: polydispersity index; [η]: intrinsic viscosity; FA: ferulic acid.

**Table 2:** Antioxidant capacity and total phenolic content of AX.

Sample	EC_50_	Antioxidant Capacity	Total Phenolic
	µg/mL	µmol TEAC/g AX	mg EGA/g
AX	332.96 ± 4.08 ^a^	32.09 ± 4.77 ^c^	11.67 ± 0.37 ^a^
AX-1	225.17 ± 3.05 ^c^	44.30 ± 2.27 ^a^	11.84 ± 0.53 ^a^
AX-2	270.99 ± 8.75 ^b^	39.59 ± 0.24 ^b^	12.20 ± 0.24 ^a^

AX = Untreated AX; AX-1 = AX treated at 25% power for 15 min; AX-2 = AX treated at 50% power for 15 min. All values represent means ± SD of three replicates. Different letters (^a–c^) in the same column differ significantly (*p* < 0.05).

**Table 3:** Protection factor values of AX on oxidative stability of soybean oil.

Treatment	PF ^1^		
	Concentration (% *w*/*v*)		
	0.01	0.10	0.25
AX	1.01 ± 0.02 ^a^	1.33 ± 0.02 ^b^	1.83 ± 0.03 ^c^
AX-1	1.31 ± 0.01 ^c^	1.35 ± 0.02 ^b^	1.46 ± 0.00 ^b^
AX-2	1.17 ± 0.01 ^b^	1.15 ± 0.01 ^a^	1.29 ± 0.05 ^a^
0.02% (*w*/*v*) TBHQ ^2^	5.24 ± 0.52 ^e^		
0.02% (*w*/*v*) Tocopherol	3.18 ± 1.10 ^d^		

^1^ PF = protection factor. ^2^ TBHQ = tertbutylhydroquinone. Values are presented as means ± SD of three replicates. Different letters (^a–e^) in the same column differ significantly (*p* < 0.05).
